# P-1962. SEAL: Serology-based Assessment of Evusheld in Lung Transplant Recipients

**DOI:** 10.1093/ofid/ofae631.2121

**Published:** 2025-01-29

**Authors:** Kevin P FitzGerald, Gordana Simeunovic, Jose Morillas, James Polega, Heather Brooks, Rebecca P Emery, Joshua D Donkin

**Affiliations:** Corewell Health/Michigan State University, Grand Rapids, Michigan; Corewell Health/ Michigan State University, grand rapids, Michigan; Corewell Health West, Grand Rapids, Michigan; Corewell Health, Grand Rapids, Michigan; OSF HealthCare, Rockford, Michigan; Corewell Health West, Grand Rapids, Michigan; Corewell Health/MSU College of Human Medicine, Grand Rapids, Michigan

## Abstract

**Background:**

The primary strategy to combat severe COVID-19 infection in Lung Transplant Recipients (LTRs) is prevention through vaccination or the use of monoclonal antibodies such as Evusheld (tixagevimab/cilgavimab), for pre-exposure prophylaxis. Data on Evusheld efficacy overall and particularly in relation to the serological status of LTRs is scarce. We evaluated treatment outcomes in LTRs within 1 year after treatment with Evusheld based on their serological status before Evusheld administration.

Study Design

**Figure:**
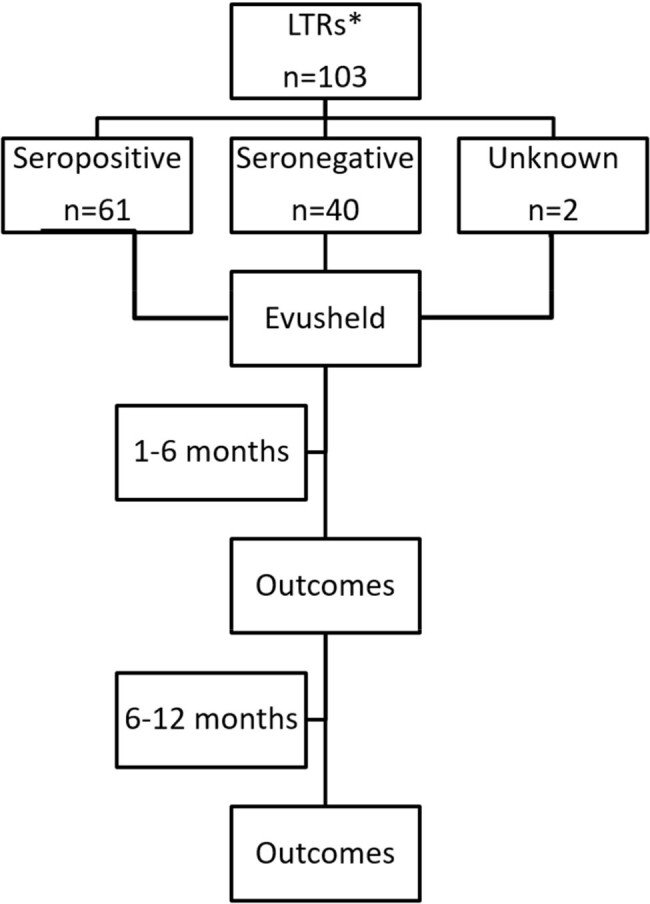

Of the 103 Lung Transplant recipients (LTRs*) who received Evusheld, 101 patients had been tested for anti-spike antibodies against SARS-CoV-2 prior to receiving Evusheld. 61 patients tested positive (seropositive) and 40 tested negative (seronegative). All patients were followed for 12 months. Outcomes were evaluated after the first 6 months when Evusheld was active, and after the second 6 months when Evusheld either was not used or had lost its efficacy against circulating strains.

**Methods:**

This is a retrospective cohort analysis of 103 LTRs treated with Evusheld between January 27 and May 25, 2022, at Corwell Health in West Michigan. LTRs were classified into seropositive (SeroPos) and seronegative (SeroNeg) groups based on the presence of anti-spike antibodies against SARS-CoV-2 before Evusheld administration and evaluated after the first 6 months, when Evusheld was active, and after the second 6 months, when Evusheld was ineffective against circulating strains (Figure 1), for outcomes listed in Figure 2.

**Figure:**
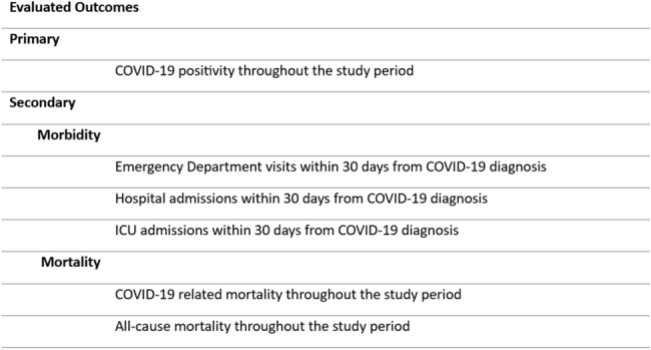

We identified patients with positive COVID-19 test, either antigen or PCR. Morbidity was assessed through the rate of ED visits, hospital, and ICU admissions within 30 days from COVID-19 diagnosis. Mortality was assessed through COVID-19-related and all-cause deaths throughout the study period.

**Results:**

Of 103 LTRs, 97% were vaccinated, 19% had COVID-19 and 59% were SeroPos before receiving Evusheld. Compared with SeroNeg LTRs, SeroPos LTRs were more likely to have had prior COVID-19 infection (22.95% v 12.5%, p=0.19). Vaccination rate was similar in both groups (98.36 vs 95%, p=0.56). SeroNeg LTRs were overall more likely to contract COVID-19, with a stable infection rate of 15% across the whole study period. In SeroPos LTRs, the positivity rate doubled in the 2nd 6 months compared with the 1st 6 months (6.5% vs. 13%, p=0.24). There was no difference in the disease severity between SeroPos and SeroNeg patients at any point (Figure 3). Overall, LTRs were less likely to contract COVID-19, be admitted to the hospital/intensive care unit, or die during the first 6 months when Evusheld was effective, compared with the second 6 months, but the difference was not statistically significant. (Figure 4).

Characteristics and outcomes in seropositive and seronegative groups

**Figure:**
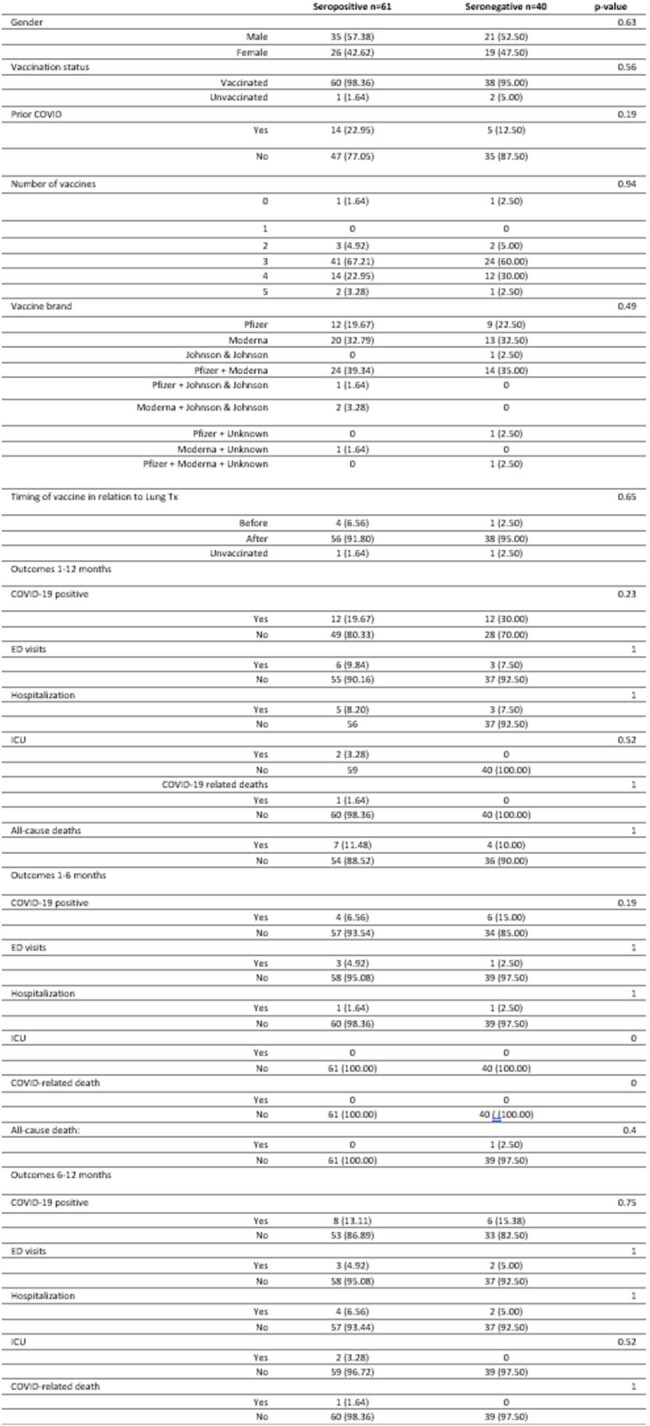

**Conclusion:**

Overall COVID-19 infection outcomes in LTRs had tendency to be better with Evsuheld use. SeroNeg LTRs were more likely to contract COVID-19 with and without Evusheld protection but serological status may not correlate with the disease severity. Our findings suggest that prior COVID-19 infection may induce a stronger immune response than the vaccine in LTRs.

Outcome in all Lung Transplant Recipients Treated with Evusheld

**Figure:**
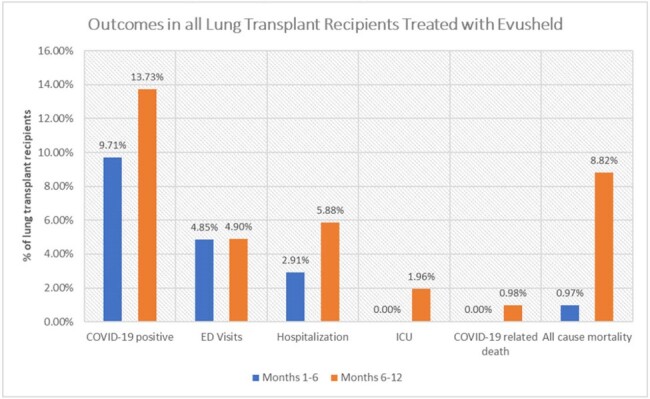

Lung transplant recipients were more likely to contract COVID-19 (13.73% vs. 9.71%, p=0.37), visit Emergency Department (ED (4.9% vs. 4.85%, p=1), get admitted to hospital (5.88% vs. 2.91%, p=0.33) or Intensive Care Unit (ICU) (1.96% vs. 0%, p=0.25), within the second 6 months compared with the first 6 months. All-cause mortality was significantly higher in the second 6 months (8.82% vs. 0.97%, p= 0.0097) but there was no difference in COVID-19 related deaths (0.98% vs. 0%, p=0.5).

**Disclosures:**

All Authors: No reported disclosures

